# NMR Structure of Integrin α4 Cytosolic Tail and Its Interactions with Paxillin

**DOI:** 10.1371/journal.pone.0055184

**Published:** 2013-01-31

**Authors:** Geok-Lin Chua, Alok Tanala Patra, Suet-Mien Tan, Surajit Bhattacharjya

**Affiliations:** School of Biological Sciences, Nanyang Technological University, Singapore, Singapore; George Washington University, United States of America

## Abstract

**Background:**

Integrins are a group of transmembrane signaling proteins that are important in biological processes such as cell adhesion, proliferation and migration. Integrins are α/β hetero-dimers and there are 24 different integrins formed by specific combinations of 18 α and 8 β subunits in humans. Generally, each of these subunits has a large extracellular domain, a single pass transmembrane segment and a cytosolic tail (CT). CTs of integrins are important in bidirectional signal transduction and they associate with a large number of intracellular proteins.

**Principal Findings:**

Using NMR spectroscopy, we determined the 3-D structure of the full-length α4 CT (Lys968-Asp999) and characterize its interactions with the adaptor protein paxillin. The α4 CT assumes an overall helical structure with a kink in its membrane proximal region. Residues Gln981-Asn997 formed a continuous helical conformation that may be sustained by potential ionic and/or hydrogen bond interactions and packing of aromatic-aliphatic side-chains. ^15^N-^1^H HSQC NMR experiments reveal interactions of the α4 CT C-terminal region with a fragment of paxillin (residues G139-K277) that encompassed LD2-LD4 repeats. Residues of these LD repeats including their adjoining linkers showed α4 CT binding-induced chemical shift changes. Furthermore, NMR studies using LD-containing peptides showed predominant interactions between LD3 and LD4 of paxillin and α4 CT. Docked structures of the α4 CT with these LD repeats suggest possible polar and/or salt-bridge and non-polar packing interactions.

**Significance:**

The current study provides molecular insights into the structural diversity of α CTs of integrins and interactions of integrin α4 CT with the adaptor protein paxillin.

## Introduction

Integrins are cell adhesion receptors that regulate cell migration, cytoskeletal remodeling, and gene expression [1], [2], [3]. In humans, 24 integrins are formed by specific non-covalent pairing of 18 α and 8 β subunits [4]. Each subunit has a large extracellular region that is involved in ligand-binding and a single-pass transmembrane segment for the transmission of allostery across the cell’s plasma membrane. This is followed by a short cytosolic tail (CT) except that of the integrin β4 subunit [5]. Integrin CTs associate with cytoskeletal, adaptor, and signaling proteins, which allow cells to communicate extracellular biochemical and mechanical signals with intracellular signaling pathways [4], [6], [7].

Integrin α4β1 (CD49dCD29; very late activation antigen, VLA-4) is expressed abundantly on leukocytes except neutrophils. The other leukocyte integrin having the same α subunit is α4β7. Integrin α4β1 binds to the alternatively spliced connecting segment -1 (CS-1) in fibronectin, activated endothelium-expressed vascular cell adhesion molecule-1 (VCAM-1), and osteopontin [8], [9], [10]. In addition to fibronectin and VCAM-1, integrin α4β7 binds mucosal addressin cell adhesion molecule-1 (MadCAM-1) that is expressed on high endothelial venules of Peyer’s patches and in gut-associated lymphoid tissues, allowing the targeting of lymphocyte subsets to these sites [11], [12]. Apart from the widely reported β2 integrins [5], both α4 integrins mediate rolling and firm adhesion of lymphocytes on endothelium [13], [14]. VCAM-1-engaged integrin α4β1was shown to up-regulate integrin αLβ2-mediated leukocyte adhesion, suggesting crosstalk between integrins [15], [16]. The importance of α4 integrins is also underscored by embryonic lethality observed in mice that were homozygous for integrin α4 gene ablation [17]. Subsequently, the use of chimeric mice provided evidence that α4 integrins are also essential for the normal development of T and B lymphocytes in the bone marrow [18]. Hence, α4 integrins are attractive targets for the development of therapeutics for inflammatory diseases. The drug Natalizumab, which is a humanized function-blocking mAb that binds the α4 subunit, has been used for the treatment of autoimmune diseases such as multiple sclerosis and Crohn disease [19], [20].

Integrin α4β1 mediates chemotactic and haptotatic cell migration on VCAM-1 whereas replacing the α4 CT with that of either integrin α2 or α5 induces focal complex formation with a concomitant increase in the strength of cell adhesion [21]. Hence, intracellular signaling events derived from integrin α4β1and other β1 integrins are different even though they have a common β1 subunit, suggesting the importance of the α subunits in integrin signaling. A seminal report by Liu et al., identified α4 CT, but not CTs of αIIb, α3A, α5, α6 and β1 integrins, as a binding partner of the adaptor protein paxillin [22]. Using fragments of integrin α4 CT and paxillin, the interaction sites were mapped to E983-Y991 in α4 and A176-D275 in paxillin [23], [24]. Interestingly, integrin α9 CT has also been shown to interact with paxillin possibly because of the sequence homology between α4 and α9 CTs [25], [26].

Paxillin is a widely expressed 68-kDa adaptor protein that contains five leucine-rich LD repeats and four LIM domains in its N- and C-terminal halves, respectively. Its LIM3 and LIM4 domains have been shown to interact with protein tyrosine phosphatase (PTP)-PEST [27], [28]. PTP-PEST regulates the activity of p130Cas (Crk-associated-substrate) that is involved in adhesion mechano-sensing and cell migration [29], [30]. The N-terminal region of paxillin that contains the LD repeats supports the binding of many proteins, including Src, Csk, vinculin, focal adhesion kinase (FAK) and proline rich tyrosine kinase 2 (Pyk2) [31], [32]. The stretch of amino acids A176-D725 in paxillin that binds integrin α4 CT encompasses the LD3 and LD4 repeats [24].

Paxillin-integrin α4 and -integrin α9 interactions inhibit cell spreading and lamellipodia formation [22], [26], [33]. Mutating Tyr991 to Ala in the α4 CT disrupts its interaction with paxillin and Jurkat T cells expressing this mutated integrin showed extensive spreading on VCAM-1 [22]. Transgenic mice homozygous for α4 Y991A had reduced number of Peyer’s patches and exhibited poor recruitment of leukocytes in thioglycollate-elicited peritonitis compared with wild-type mice [34]. Paxillin binding to α4 CT is also regulated by post-translational modification of the latter. Phosphorylation of Ser988 in α4 CT inhibits its binding to paxillin [35]. Notably, α4 CT with pSer988 was reported to be lacking at the trailing region of migrating cells [36]. Taken together, these data establish a role of paxillin in regulating adhesion sites turnover that is critical in cell migration [32].

Based on high throughput screening, a small molecule has been identified to disrupt the α4 CT-paxillin interaction and it reduced leukocyte recruitment to sites of inflammation in mice [37]. Conceivably, structural determination of α4 CT-paxillin interaction will provide valuable information to understand how the interaction is regulated and pave the way for the development of novel therapeutics. In this study, we determine the conformation of the full-length integrin α4 CT in aqueous solution and analyze its interactions with recombinant paxillin (residues G139-K277 encompassing LD2-LD4) and synthetic peptides containing LD2, LD3 or LD4. We provide evidence that the C-terminal region of integrin α4 CT adopts a helical conformation and it is involved in binding to the LD3 and LD4 repeats of paxillin.

## Results and Discussion

### NMR Studies of Integrin α4 CT

**Figure 1:**
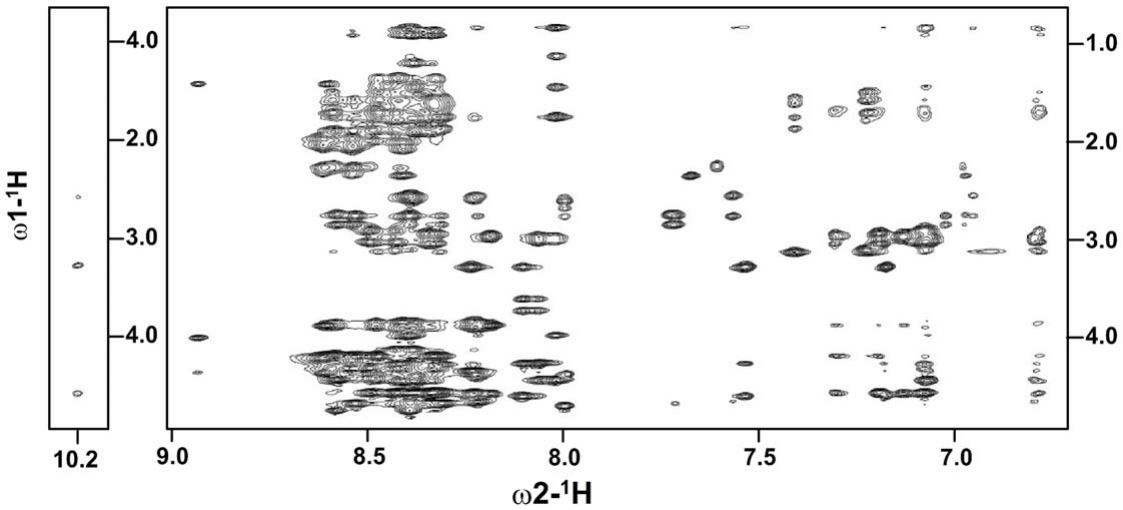
Folded conformations of α4 CT. A section of the two-dimensional ^1^H-^1^H NOESY spectrum of α4 CT in aqueous solution showing NOE contacts among low-field resonances (6.5 ppm-9.0 ppm) with up-field resonances (0.8 ppm- 4.5 ppm) (right panel). NOE contacts from the low-field shifted N^ε^H resonance, 10.2 ppm, of the residue W22 with backbone and sidechain aliphatic proton resonances (left panel). NOESY spectra were acquired in aqueous solution, pH 5.6, 278 K.

**Figure 2:**
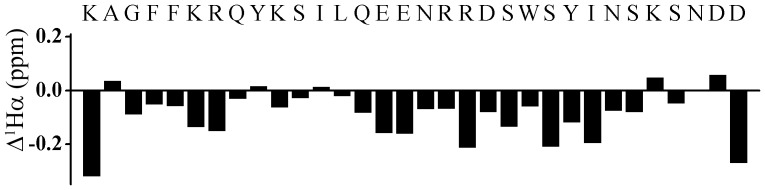
Secondary structure of the α4 CT from chemical shift deviations. Bar diagrams representing deviation of CαH chemical shifts from random coil values for amino acid residues of α4 CT in aqueous solution.

**Figure 3:**
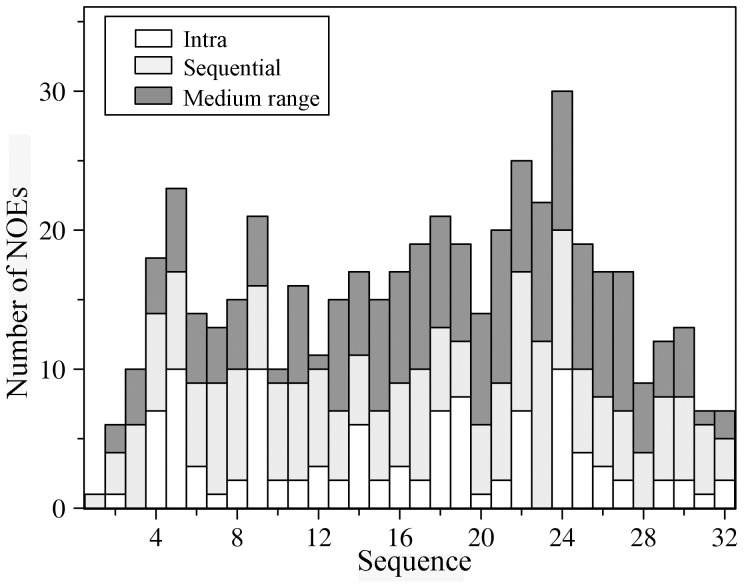
Summary of NOE contacts of the α4 CT. Bar diagram summarizing type (intra, sequential, medium-range) and number of NOE contacts observed for each amino acid of the α4 CT in aqueous solution.

3-D structures of the CTs of αIIb, αM and αX integrins were determined by NMR spectroscopy in lipid micelles by having a myristoyl chain covalently-linked to the N-terminus of each CT [38], [39], [40]. The micelle anchoring property of the myristoyl group that mimics the transmembrane segment imparts conformational stabilization to the α CTs [38], [39], [40]. We have attempted a similar strategy to solve the NMR structure of α4 CT in DPC lipid micelles. However, NMR spectra of the myristoylated α4 CT was found to be extremely broad, precluding structural characterization under such conditions (data not shown). It is noteworthy that apart from the conserved membrane proximal GFFKR motif, the primary structure of α4 CT is unique from other α CTs (Supplementary Figure S1). Interestingly, α4 CT shows well dispersed NMR spectra in free aqueous solution. Figure 1 shows a section of the 2-D NOESY spectrum of α4 CT, at 278 K, delineating NOE connectivites among the down-field shifted (6.5–9.0 ppm), amide and aromatic, proton resonances with the up-field shifted (0.9–4.5 ppm) aliphatic proton resonances. NOE connectivities were also detected from the N^ε^H proton of the residue W22, resonating at 10.2 ppm, with the aliphatic protons (Figure 1, left panel). These NOE cross-peaks potentially indicate populated folded conformations of the α4 CT in aqueous solution. The sequence-specific resonance assignments of α4 CT was achieved by combined analyses of 2-D TOCSY and 2-D NOESY spectra. The secondary chemical shifts or deviation from random coil values of C^α^H resonances of each amino acid of α4 CT are shown in Figure 2. Helical conformations appear to be populated for the stretch of C-terminus residues, L13-S27, and membrane proximal N-terminal segment, residues G3-R7, of α4 CT as indicated by the negative deviation for C^α^H chemical shifts. The secondary chemical shifts are less pronounced for other C-terminal residues including K28-D32, indicating a lack of preferred secondary conformations (Figure 2). Further, analyses of 2-D NOESY spectra of α4 CT revealed sequential and medium range NOEs (C^α^H/NH: i to i+2, i+3 and i+4) diagnostic of helical conformations for most of the residues, L13-N30, at the C-terminus (Figure 3). Helical type medium range NOEs were also detected for the N-terminal membrane proximal region.

### Three-dimensional Structure of Integrin α4 CT

**Figure 4:**
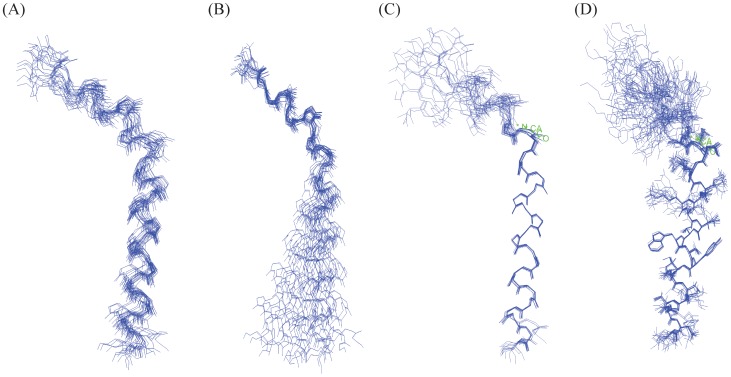
Three-dimensional structure of the α4 CT. (A) Superposition of backbone atoms (N, Cα, C′) of twenty lowest energy conformers of the α4 CT for residues A2-D31. (B) Superposition of backbone atoms (N, Cα, C′) of twenty lowest energy conformers of the α4 CT for N-terminal residues A2-L13. (C-D) Superposition of backbone atoms (N, Cα, C′) of twenty lowest energy conformers of the α4 CT for C-terminal residues Q14-D31. Superposition of the sidechains of residues Q14-D31 are also shown (D). Figures were generated by INSIGHT II.

**Figure 5:**
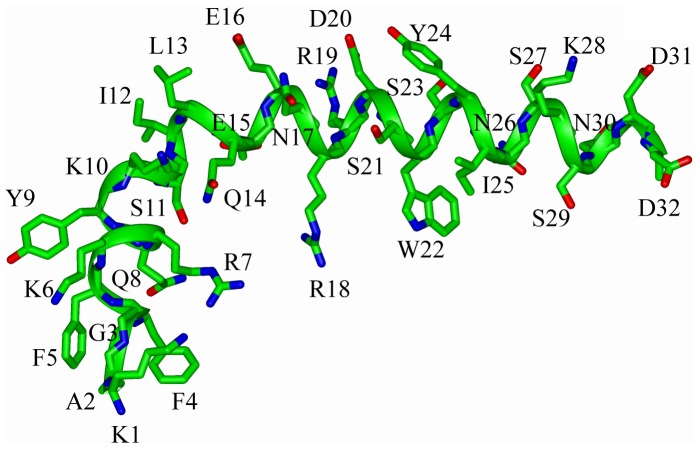
Inter side-chain interactions and disposition of the α4 CT helical structure. Ribbon representations of the helical structure of α4 CT. Sidechains are shown as sticks. The helical structure contains a bent at the end of N-terminal half. A number of ionic and/or hydrogen bonding interactions are probable for the C-terminal region of the α4 helix. Figure was generated by INSIGHT II.

**Figure 6:**
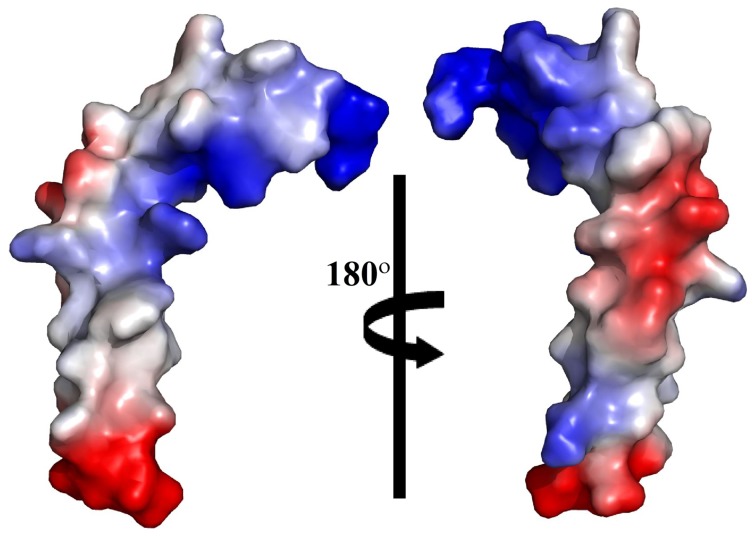
Electrostatic surface potential of α4 CT. A representative structure is shown for before and after 180° rotation along the z-axis. Surfaces in red, blue and white represent, respectively, negatively charged, positively charged and neutral residues. The figure was generated by PyMOL.

An ensemble of conformations of α4 CT were obtained from 300 NOE driven distance constraints based on 107 intra-residue, 95 sequential and 98 medium range NOEs (Table 1). Figure 4 shows superposition of the backbone atoms of the structural ensemble of α4 CT for residues A2-D31 (panel A), residues A2-L13 (panel B) and residues Q14-D31 (panels C-D). Higher root mean square deviation (RMSD) values for the backbone and all heavy atoms can be seen for the superposed structures while including residues A2-D31 of α4 CT (Figure 4A, Table 1). However, RMSD values are found to be lower for the individual segment of the N- and C-termini of the molecule (Table 1). Notably, the C-terminal region, residues Q14-D31, of α4 CT demonstrates a well-defined backbone and side-chain topology in comparison to the N-terminal region (Table 1, Figure 4). The α4 CT assumes a bend or kinked helical conformation in free solution (Figure 5). The membrane proximal region demarcates a conserved helical conformation that is connected to the C-terminal helix through a bend formed by residues S11-L13. The present study reveals a different conformation of the α4 CT compared with CTs of other integrins. Three-dimensional structures of the CTs of αIIb (20-residue), αM (24-residue) and αX (35-residue) are characterized by an N-terminus membrane proximal helix followed by a C-terminal loop [38], [39], [40]. The tertiary topologies of these α CTs are stabilized by long-range packing between the loop and the N-terminal helix. On the other hand, the longer CT (57-residue) of αL integrin assumes a packed structure consisting of three helices [41]. By contrast, the C-terminal region of α4 CT adopts a helical conformation that does not show any long-range packing interactions with the membrane proximal helix (Figure 5). The C-terminal helix of α4 CT can potentially be stabilized by a number of polar interactions, ionic and/or hydrogen bonds, by the sidechains of residues E16-R19, K28-D31, N17-S21, D20-S23 and N26-S29 (Figure 5). In addition, the indole ring of residue W22 is in a close proximity with the aliphatic sidechain of residue I25 and guanidinium sidechain of residue R18, implying probable cation-π and/or non-polar packing interactions. The helical structure of α4 CT has patches of negatively and positively charged surfaces for its C-terminal region, whereas the N-terminal region is largely positively charged (Figure 6).

**Table 1:** Summary of structural statistics of the twenty lowest energy structures of α4 CT in aqueous solution.

**Distance restraints**	Intra-residue(|i – j| = 0)	107
	Sequential(|i – j| = 1)	95
	Medium range (2≤|i-j|≤4)	98
	Total	300
**Dihedral angle constraints (Φ)**		31
**Constraints violations**	Average NOE violation (Å)	0.24
	Maximun NOE violation (Å)	≤0.24
**Deviation from mean structure**	Backbone atoms (N, Cα, C′) (Å)	1.15 (N-terminal: 0.85, C-terminal: 0.45)
	Heavy atoms (Å)	1.86 (N-terminal: 1.52, C-terminal: 0.95)
**Ramachandran plot analysis**	% residues in the most favorable region	93
	% residues additionally allowed region	7
	% residues in the generously allowed region	0
	% residues in the disallowed region	0

### Mapping Residues of PaxLD2-LD4 that Interact with the Integrin α4 CT by ^15^N-^1^H HSQC

**Figure 7:**
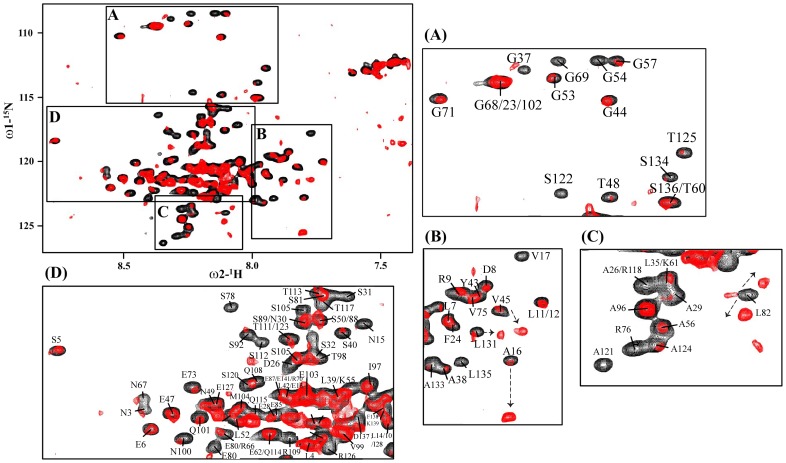
Determination of interactions between ^15^N-labeled PaxLD2-LD4 and α4 CT by ^15^N-^1^H HSQC NMR. ^15^N-^1^H HSQC and selected sections (panels A-D) of ^15^N-^1^H HSQC spectrum of ^15^N-labeled PaxLD2-LD4 in the absence of (black contour) and in the presence of (red contour) unlabelled α4 CT at a ratio of 1∶2 (PaxLD2-LD4:α4 CT).

For interactions studies, we have expressed and purified full-length α4 CT and an N-terminus fragment of paxillin (residues G139-K277 or PaxLD2-LD4) that encompassed LD2, LD3 and LD4. The N-terminus region of paxillin that contains the LD repeats has been shown to bind the α4 CT [24]. The ^15^N-^1^H HSQC spectra of PaxLD2-LD4 and α4 CT were assigned using standard triple resonance experiments. An overlay of ^15^N-^1^H HSQC spectra of the ^15^N-labeled PaxLD2-LD4 in the absence of (black contour) and in the presence of (red contour) two-fold excess of unlabeled α4 CT is shown (Figure 7). There are significant perturbations in the HSQC spectra of PaxLD2-LD4 upon addition of α4 CT, which suggest interactions. Notably, a large number of HSQC cross-peaks of PaxLD2-LD4 demonstrate loss in intensity as a consequence of complex formation (Figure 7 panels A-D). In addition, new HSQC peaks are observed close to the ^15^N-^1^H HSQC peaks of residues A16, V45 and L131 (panel B), residue L82 (panel C) and residue S112 (panel D). Because of the extensive resonance overlapping in the ^15^N-^1^H HSQC spectra, arising from a preponderance of similar residues in the amino acid sequence of paxillin fragment, binding induced changes are assessed only for well separated ^15^N-^1^H HSQC cross-peaks. The ^15^N-^1^H HSQC cross-peaks of Gly and Ser/Thr residues are well separated from others as a result of intrinsic upfield shift in ^15^N chemical shift (Figure 7A). The intensity of the ^15^N-^1^H HSQC peaks are significantly diminished for residues G37, G54, G57, G69, S122, S134 and T125. These Gly residues are situated at the linker region between the LD repeats. Similar perturbations can be seen for residues in the LD repeats of PaxLD2-LD4 (Figure 7). Residues of PaxLD2-LD4 showing binding induced resonance perturbations are listed in Table 2. Interestingly, resonance perturbation can be seen for residues located in all the three LD repeats and those in the linker regions. In addition, more residues are perturbed in LD3 and LD4 repeats in comparison to the LD2 repeat. The ^15^N-^1^H HSQC cross-peaks of residues E6, L7, L11, L12 of LD2 repeats are not significantly affected in the presence of the α4 CT (Figures 7B and 7D). However, changes observed for the ^15^N-^1^H HSQC cross-peaks of the linker residues of PaxLD2-LD4 are rather intriguing (Table 2). Collectively, we surmise that binding to the 32-residue α4 CT induces global conformational changes of the entire sequence of PaxLD2-LD4. These are likely to yield ^15^N-^1^H HSQC spectral changes away from the binding interface [42].

**Table 2:** Residues of PaxLD2-LD4 showing changes in intensity (>70%) and/or chemical shifts in ^15^N-^1^H HSQC spectra after addition of α4 CT.

Structural Location	Residue
LD2	D8, R9, N15, A16, V17
Linker between LD2-LD3	D27, E28, A29, S31, S32, G37, A38, S40, T48, G53, G54, G57, N67, G69
LD3	E73, V75, R76, S78, E80, S81, L82, E85
Linker between LD3-LD4	S92, T98, N100, S105, Q108, R109, T111, S112, S120, A121, S122, S123, A124,
LD4	T125, R126,L131, A133, S134, L135, D137

a G-S-N-**L-S^5^-E-L-D-R-L^10^-L-L-E-L**-**N^15^-A-V**-Q-H-N^20^-P-P-G-F-P^25^-A-D-E-A-N^30^-S-S-P-P-L^35^-P-G-A-L-S^40^-P-L-Y-G-V^45^-P-E-T-N-S^50^-P-L-G-G-K^55^-A-G-P-L-T^60^-K-E-K-P-K^65^-R-N-G-G-R^70^-G-**L-E-D-V^75^-R-P-S-V-E^80^-S-L-L-D-E^85^-L-E**-S-S-V^90^-P-S-P-V-P^95^-A-I-T-V-N^100^-Q-G-E-M-S^105^-S-P-Q-R-V^110^-T-S-T-Q-Q^115^-Q-T-R-I-S^120^-A-S-S-A-**T^125^-R-E-L-D-E^130^-L-M-A-S-L^135^-S-D**-F-K.

**a**:Amino acid sequence of PaxLD2-LD4. The LD repeats are bold faced.

### Mapping Residues of Integrin α4 CT that Interact with PaxLD2-LD4 by ^15^N-^1^H HSQC

**Figure 8:**
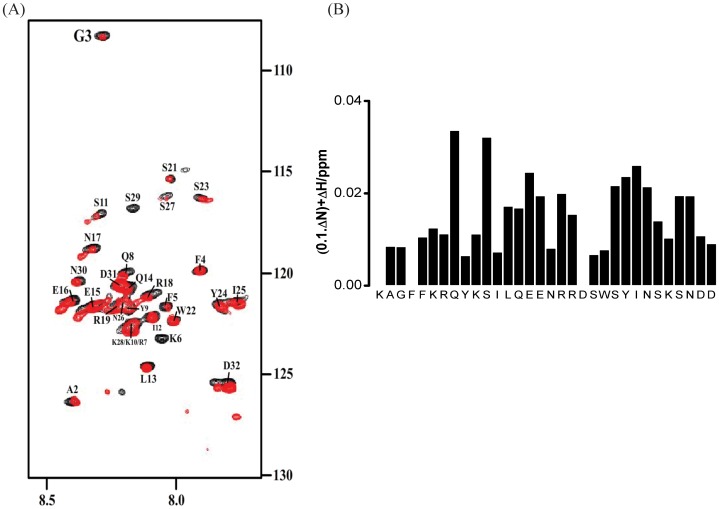
Determination of interactions between ^15^N-labeled α4 CT and PaxLD2-LD4 by ^15^N-^1^H HSQC NMR. (A) ^15^N-^1^H HSQC spectrum of ^15^N-labeled α4 CT in the absence of (black contour) and in the presence of (red contour) unlabelled PaxLD2-LD4 at a ratio of 1∶2 (α4 CT:PaxLD2-LD4). (B) A bar diagram summarizing combined chemical shift changes of ^15^N and ^1^HN resonances of the α4 CT as a function of amino acids.

^15^N-^1^H HSQC spectra of ^15^N-labeled α4 CT in the absence of (black contour) and in the presence of (red contour) unlabeled PaxLD2-LD4 are shown (Figure 8). Addition of PaxLD2-LD4 caused chemical shift and/or intensity changes for several ^15^N-^1^H HSQC cross-peaks of α4 CT, indicating binding (Figure 8A). From the combined chemical shift changes of ^15^N and ^1^HN nuclei of α4 CT, residues Q8, S11, E15, E16, S23, Y24, I25, N26, S29, N30 and D32 exhibit higher chemical shift changes (Figure 8B). In addition, ^15^N-^1^H HSQC cross-peaks of residues K6, S11, S27 and S29 become less intense in the presence of α4 CT (Figure 8A). This may result from the broadening of resonances as intermediate chemical exchange between the free and bound states of the molecule occurs. Comparing the two sets of ^15^N-^1^H HSQC data we obtained for interactions between PaxLD2-LD4 with α4 CT, there are more resonance perturbations detected in PaxLD2-LD4 than α4 CT. This suggests that PaxLD2-LD4 undergoes larger conformational changes compared with α4 CT when they interact. However, chemical shift changes of α4 CT occurred upon binding with PaxLd2-LD4 were reproducible in repeated measurements. As can be seen, PaxLD2-LD4 induced resonance perturbations mainly from residues in the C-terminal helix of α4 CT (Figure 8). Limited resonance perturbations of α4 CT membrane proximal residues K1-K10 were detected (Figure 8B). These results demonstrate that the C-terminal region of α4 CT is primarily responsible for its interactions with PaxLD2-LD3. This is consistent with the finding of a previous study that identified involvement of the C-terminal region of α4 CT for binding to paxillin [23].

### Binding of Integrin α4 CT with LD-containing Peptides of Paxillin

**Figure 9:**
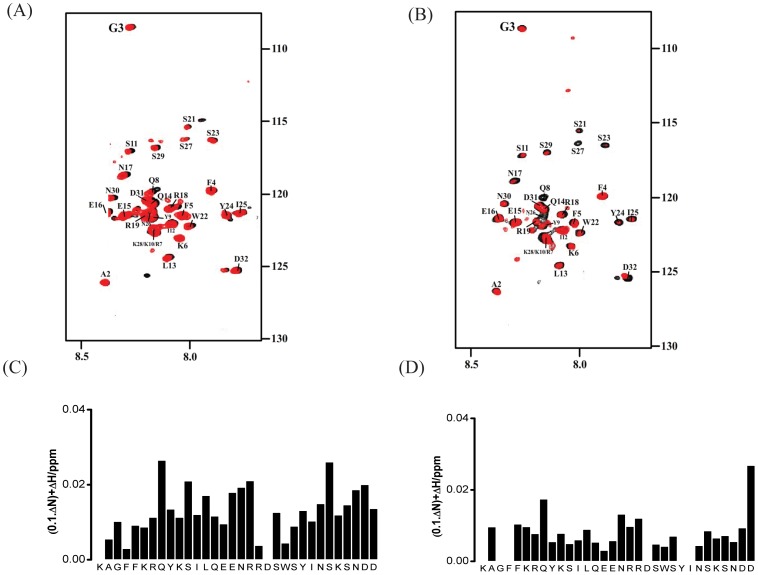
Determination of interactions between ^15^N-labeled α4 CT and LD repeats of paxillin by ^15^N-^1^H HSQC NMR. ^15^N-^1^H HSQC spectrum of ^15^N labeled α4 CT in the absence of (black contour) and in the presence of (red contour) unlabelled peptide-containing LD3 (A) or unlabelled peptide-containing LD4 (B) at a ratio of 1∶4 (α4 CT:peptides). Bar diagrams summarizing combined chemical shift changes of ^15^N and ^1^HN resonances of the α4 CT as a function of amino acids in the presence of LD3 (C) or LD4 (D).

Atomic-resolution structures have been determined for LD repeats of paxillin in complex with well-folded FAT domain of FAK and with CH domain of the adaptor protein parvins [43], [44], [45], [46], [47]. We therefore examine the binding interactions of three synthetic peptide fragments containing LD2 (NLSELDRLLLELNAVQHN), LD3 (VRPSVESLLDELESSVPSPV) and LD4 (ATRELDELMASLSDFKFMAQ), with the α4 CT. ^15^N-^1^H HSQC spectra overlays of α4 CT in the absence of (black contour) and in the presence of (red contour) LD3 (Figure 9A) and LD4 (Figure 9B) are shown. Addition of LD3-containing peptide caused chemical shift changes only for residues Q8, S11, N17, S27, S29, N30, D31 of the α4 CT (Figures 9A and 9C). Addition of LD4-containing peptide reduced the signal intensity of ^15^N-^1^H cross-peaks of α4 CT residues Q8, S21, S27, S23, Y24, and I25, presumably occurring from conformational exchanges (Figure 9B). By contrast, ^15^N-^1^H HSQC spectra of the α4 CT were largely unaffected in the presence of LD2-containing peptide (Supplementary Figure S2). Indeed, PaxLD2-LD4 experiments have provided insights into residues corresponding to LD3 and LD4 that are affected by α4 CT interactions (Table 2). Collectively, these results suggest that α4 CT binds directly to paxillin LD3 and LD4 repeats.

### A Molecular Model of α4 CT in Complex with LD3 and LD4 Repeats of Paxillin

**Figure 10:**
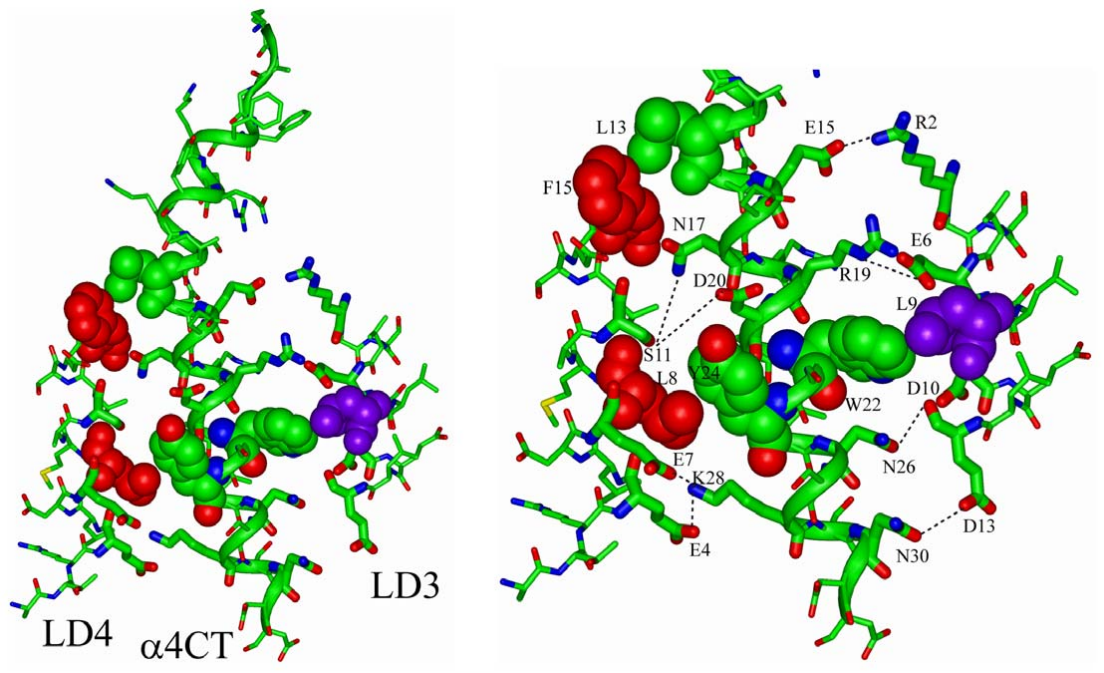
Docked structure of the α4 CT with LD3 and LD4 repeats of paxillin. The C-terminal region of the α4 CT (in green ribbon) may form interfaces with helices of LD3 (in stick) and LD4 (in stick). The interactions could be maintained by ionic and/or hydrogen bonding and van der Waals’ packing among non-polar residues (right panel). The probable packing of side-chains among the non-polar and aromatic residues, L8, F15 (in red) of LD4 with Y24, L13 (in green) of α4 CT and L9 (in purple) of LD3 with W24 of α4 CT(in green) represented by space filling. The potential ionic and/or hydrogen bond interactions are marked by broken lines. Figures were generated by INSIGHT II.

LD repeats adopt helical structures whereby the non-polar face of the helix, containing most of the Leu residues, are inserted into the binding pocket of target proteins whereas the polar face of the helix that contains acidic residues Asp/Glu remains exposed to the solvent [43], [44], [45], [46], [47]. Because the C-terminus of the α4 CT is highly polar, we generated a docking model of α4 CT with LD3 and LD4 repeats of paxillin by maximizing potential polar interactions in the complex (Figure 10). In the docked structure, helices of LD3 and LD4 repeats are arranged in a sequential orientation with the α4 CT helix, whereas LD3 helix is parallel and LD4 helix orients in an anti-parallel fashion (Figure 10). There are a number of potential ionic, hydrogen bond and non-polar packing interactions that may sustain the α4 CT and paxillin LD repeats complex (Figure 10, left panel). The interface between LD3 repeat and α4 CT can potentially be stabilized by salt bridges formed by side-chains of residues R2 and E6 of LD3 and residues E15/E16 and R19 of α4 CT, respectively. In addition, sidechains of residues D10 and D13 of the LD3 are in a close proximity with the sidechains of N26 and N30 of α4 CT, suggesting interactions via hydrogen bonds. The non-polar sidechain of residue L9 of LD3 is partially exposed and can make van der Waals’ packing with the aromatic sidechain of residue W22 of α4 CT. The LD4 helix docks onto the opposite face of the α4 helix. The helix-helix packing can be maintained by potential ionic and/or hydrogen bonding interactions among the side-chains of residues E4, E7, S11 of LD4 with the side-chains of residues K28, D20 and S21 of α4 CT. There are also packing interactions of residues L8 and F15 of LD4 with Y24 and L13 of α4 CT, respectively. The model that we proposed herein for α4 CT in complex with LD3 and LD4 of paxillin can be supported by experimental findings in which mutating α4 CT residue E16 (or E983) or residue Y24 (or Y991) to Ala disrupted the binding of α4 CT to paxillin [23]. Further, residue S21 (or S988) of α4 CT, located at the interface of the complex, was known to modulate paxillin binding due to phosphorylation [35].

### Conclusions

Our study provides for the first time the atomic structure of integrin α4 CT. It also provides molecular insights into interactions between α4 CT and LD repeats of paxillin. Unlike CTs of αIIb, αM and αX integrins that all have an N-terminal helix followed by a C-terminal loop, the α4 CT adopts a helical C-terminal region that is involved in paxillin binding. Conceivably, sequence and structural variations of α CTs of integrins can contribute toward recruiting specific signaling proteins. Finally, our proposed model of α4 CT in complex with LD3 and LD4 of paxillin will be useful for the design and testing of small molecules that can disrupt this interaction and therefore potentially anti-inflammatory.

## Materials and Methods

### Synthetic Peptides

The sequence of α4 cytoplasmic tail from residues Lys968 to Asp999 (^968^KAGFFKRQYKSILQEENRRDSWSYINSKSNDD^999^) is re-numbered from 1–32 for ease of reference. Additionally, the paxillin region Gly139 to Lys277 or PaxLD2-LD4 is also re-numbered from 1–139. All synthetic peptides of α4 CT and those containing paxillin LD repeats (LD2: NLSELDRLLLELNAVQHN; LD3: VRPSVESLLDELESSVPSPV; LD4: ATRELDELMASLSDFKFMAQ) were purchased from GL Biochem (Shanghai, China). They were further purified using a reverse phase HPLC, Waters™ connected to a C18 column (300 Å pore size, 5 µM particle size). A linear gradient of acetonitrile/water with a flow rate of 2 ml/min was used to elute the peptides, and the major peak fractions were collected and lyophilized into powder form. Mass spectrometry was used to verify molecular weights of the peptides.

### Expression and Purification of α4 CT and PaxLD2-LD4

The full length α4 CT (Lys968 to Asp999) was cloned into a pET-31b(+) vector (Novagen EMD, San Diego) with N-terminal ketosteroid (KSI) [39], [40], [41] which has a Met cleavage site inserted prior to the α4 CT sequence. The recombinant plasmid was transformed into BL21(DE3) cells. Transformed cells were cultured overnight in Luria-Bertani (LB) broth. The culture was seeded in 1∶100 volume ratio either in LB for the preparation of unlabeled proteins or in isotope-enriched M9 minimal media, containing ^15^N ammonium chloride without/with ^13^C-glucose for the production of isotope labeled samples at 37°C in a shaking incubator. IPTG (1 mM) was used to induce protein expression for 18 hours at 25°C with a shaking speed of 150 rpm. *E. coli* cells were harvested by centrifugation at 5000 rpm for 20 min, and the bacterial pellet re-suspended in a buffer containing 0.5 M NaCl, 20 mM Tris-HCl, pH 8.0. Re-suspended cells were lysed via sonication on ice to release the recombinant fusion proteins. As the KSI recombinant protein is targeted to the inclusion bodies, cell pellets were collected via centrifugation at 14000 rpm for 30 min and re-solubilized in a buffer containing 8 M urea, 0.5 M NaCl, 20 mM Tris-HCl, pH 8.0. The supernatant containing the solubilized KSI-α4 CT was affinity purified using Nickel-NTA acid (QIAGEN) beads making use of the 6-His tag that was attached to the N-terminus of KSI-α4 CT. The fusion protein was then eluted in buffer containing 8 M urea, 0.5 M imidazole, 0.5 M NaCl, 20 mM Tris-HCl, pH 8.0. The eluted fractions were pooled and dialyzed against water at 4°C for 2 days to remove the urea, causing the formation of KSI-α4 CT precipitates that were subsequently collected by centrifuging at 5000 rpm for 30 min. The KSI-α4 CT precipitates were dissolved in 70% formic acid. For every 1 mg of KSI-α4 CT, 37.5 mg of cyanogen bromide was used for the cleavage reaction. The reaction was purged by N_2_ gas and left in the dark for 22 hours. Sodium hydroxide was used to neutralize the cyanogen bromide and the solvent was removed using a rotary evaporator leaving behind a thin film of precipitate. The precipitate was dissolved in 10 mM sodium phosphate buffer, pH 6.5 and further purified using HPLC. The identity of the cleaved peptide was verified by mass spectrometry analysis.

The PaxLD2-LD4 (residues G139 to K277) was cloned into the pET24a(+) vector with an initiation Met introduced before G139. The construct also contained a C-terminal 6-His tag for affinity purification. The plasmid DNA was transformed into BL21(DE3) cells. Protein was produced, unlabeled or isotope (^15^N, ^15^N/^13^C) labeled, by IPTG induction at 18°C for 18 hours. *E. coli* cells were harvested by centrifugation at 5000 rpm, 4°C, for 20 min. The cell pellet was re-suspended in buffer containing 0.5 M NaCl, 20 mM Tris-HCl, pH 8.0 and lysed via sonication on ice to release recombinant proteins. The cell lysate was centrifuged at 14000 rpm, 4°C, for 30 min. The supernatant was collected and affinity purification of PaxLD2-LD4 performed using NTA beads. Washing steps were performed in buffer containing 20 mM imidazole, 0.5 M NaCl, 20 mM Tris-HCl, pH 8.0. Bound PaxLD2-LD4 protein was eluted in buffer containing 0.3 M imidazole, 0.5 M NaCl, 20 mM Tris-HCl, pH 8.0. Eluted proteins were dialyzed against buffer containing 150 mM NaCl, 20 mM Tris-HCl, pH 7.0 at room temperature for 1 hour. The protein was further purified using HPLC with a linear gradient of water/acetonitrile solvents.

### NMR Experiments

All NMR experiments were recorded on a Bruker DRX 600-MHz instrument equipped with an actively shielded cryo-probe. 10% Deuterium oxide and 2 mM 2,2-dimethyl-2-silapentane-5-sulfonate (DSS) was added to all NMR samples. Chemical shifts were referenced to DSS. 2D TOCSY (mixing time: 50 ms) and 2D NOESY (mixing time: 200 ms) spectra were recorded for 0.5 mM of α4 CT dissolved in water, pH 5.6 at 278 K. Raw NMR data were processed using TOPSPIN 2.1 and analyzed with SPARKY. ^15^N-^1^H HSQC spectra of α4 CT and PaxLD2-LD4 were assigned by triple resonance HNCACB and CBCA(CO)NH experiments. Triple resonance NMR experiments were carried out using doubly labeled (^15^N/^13^C) samples of α4 CT and PaxLD2-LD4 dissolved in 10 mM sodium phosphate buffer, pH 5.6, at 298K. For interactions studies, ^15^N-^1^H HSQC spectra of either ^15^N-labeled α4 CT (100 µM) or PaxLD2-LD4 (200 µM) were obtained in the presence of unlabeled binding partners at molar ratio of 1∶1 and 1∶2 in 10 mM sodium phosphate buffer, pH 6.5, 298 K.

### Structure Calculation and Modeling

NOE intensities of α4 CT NOESY spectra were qualitatively categorized into strong, medium and weak and translated to the upper bound distance limit of 2.5 Å, 3.5 Å and 5.0 Å respectively. These distance constraints were used for structure calculations using CYANA (Combined assignment and dynamics for NMR applications) 2.1 [48]. For structure calculation backbone dihedral angle (Φ) values were restricted to −30° to 120° to limit the conformational search. Of the 100 structures, 20 lowest energy structures were selected for evaluation and analyses. PROCHECK-NMR [49] was employed to evaluate the stereochemical quality of the structural ensemble and figures were prepared using PyMOL, MOLMOL, Discovery Studio Visualizer 2.0 and Insight II. Docking of α4 CT with LD peptides of paxillin was performed using Insight II software. Helical structures of LD3 and LD4 peptide fragments were constructed for docking with α4 CT. Several round of docking exercises were conducted to achieve optimal sidechain-sidechain packing interactions. The model complex was further energy minimized using discover force field to relieve short inter-atomic contacts.

## Supporting Information

Figure S1 **Comparison of primary structures of representative α and β CTs of integrins.** Alignment of amino acid sequences of CTs of α4, αX, αM, αL, αD, αIIb integrins.
(TIF)

Figure S2 **Determination of interactions between paxillin LD2 peptide and α4 CT by ^15^N-^1^H HSQC NMR.** ^15^N-^1^H HSQC spectra of α4 CT in the absence (black contour) and in the presence (red contour) of LD2 peptide.
(TIF)
