# Supplementary figures and images for: NMR Structure of Integrin α4 Cytosolic Tail and Its Interactions with Paxillin
Source: PLoS One. 2013 Jan 31;8(1):e55184. doi: 10.1371/journal.pone.0055184 (PMC3561355; doi:10.1371/journal.pone.0055184)

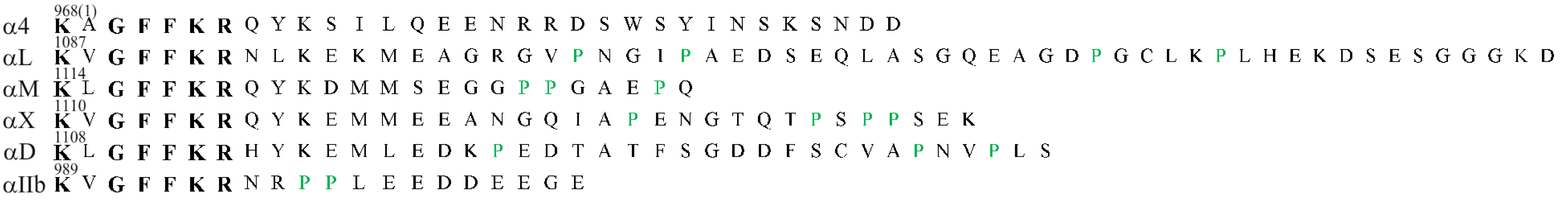

Supplement: Figure S1 — Comparison of primary structures of representative α and β CTs of integrins. Alignment of amino acid sequences of CTs of α4, αX, αM, αL, αD, αIIb integrins. (TIF) [file pone.0055184.s001.tif]

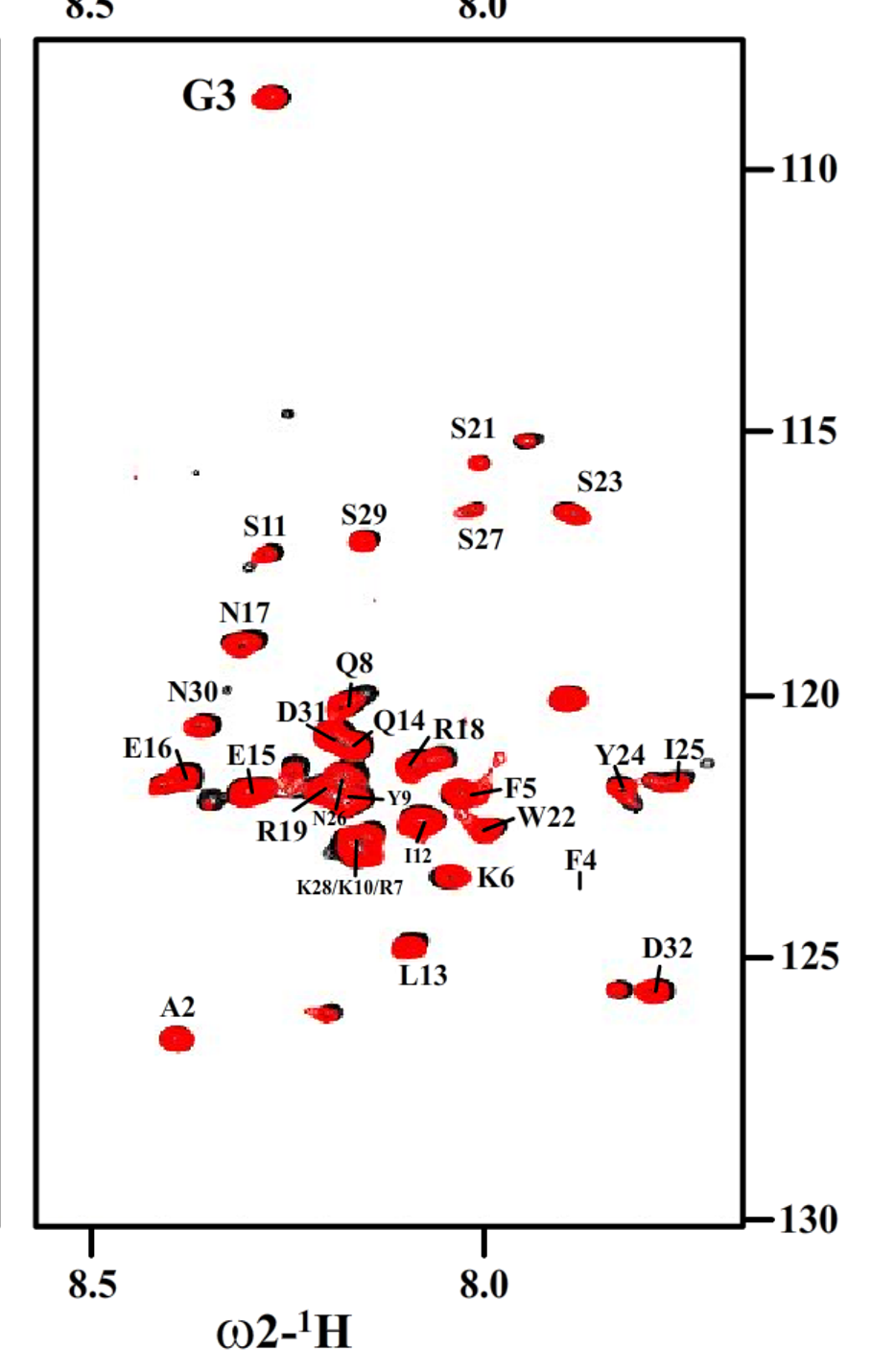

Supplement: Figure S2 — Determination of interactions between paxillin LD2 peptide and α4 CT by 15N-1H HSQC NMR. 15N-1H HSQC spectra of α4 CT in the absence (black contour) and in the presence (red contour) of LD2 peptide. (TIF) [file pone.0055184.s002.tif]
